# Dietary supplementation with inulin-propionate ester or inulin improves insulin sensitivity in adults with overweight and obesity with distinct effects on the gut microbiota, plasma metabolome and systemic inflammatory responses: a randomised cross-over trial

**DOI:** 10.1136/gutjnl-2019-318424

**Published:** 2019-04-10

**Authors:** Edward S Chambers, Claire S Byrne, Douglas J Morrison, Kevin G Murphy, Tom Preston, Catriona Tedford, Isabel Garcia-Perez, Sofia Fountana, Jose Ivan Serrano-Contreras, Elaine Holmes, Catherine J Reynolds, Jordie F Roberts, Rosemary J Boyton, Daniel M Altmann, Julie A K McDonald, Julian R Marchesi, Arne N Akbar, Natalie E Riddell, Gareth A Wallis, Gary S Frost

**Affiliations:** 1 Section for Nutrition Research, Department of Medicine, Imperial College London, London, UK; 2 Stable Isotope Biochemistry Laboratory, Scottish Universities Environmental Research Centre, Glasgow, UK; 3 Section of Endocrinology and Investigative Medicine, Imperial College London, London, UK; 4 School of Computing, Engineering and Physical Sciences, University of the West of Scotland, Paisley, UK; 5 Computational and Systems Medicine, Imperial College London, London, UK; 6 Department of Medicine, Imperial College London, London, UK; 7 Division of Integrative Systems Medicine and Digestive Disease, Department of Surgery and Cancer, Imperial College London, London, UK; 8 School of Biosciences, University of Cardiff, Cardiff, UK; 9 Division of Infectionand Immunity, University College London, London, UK; 10 Faculty of Health and Medical Sciences, University of Surrey, Guildford, UK; 11 School of Sport, Exercise and Rehabilitation Sciences, University of Birmingham, Birmingham, UK

**Keywords:** short-chain fatty acids, glucose metabolism, colonic microflora, inflammation

## Abstract

**Objective:**

To investigate the underlying mechanisms behind changes in glucose homeostasis with delivery of propionate to the human colon by comprehensive and coordinated analysis of gut bacterial composition, plasma metabolome and immune responses.

**Design:**

Twelve non-diabetic adults with overweight and obesity received 20 g/day of inulin-propionate ester (IPE), designed to selectively deliver propionate to the colon, a high-fermentable fibre control (inulin) and a low-fermentable fibre control (cellulose) in a randomised, double-blind, placebo-controlled, cross-over design. Outcome measurements of metabolic responses, inflammatory markers and gut bacterial composition were analysed at the end of each 42-day supplementation period.

**Results:**

Both IPE and inulin supplementation improved insulin resistance compared with cellulose supplementation, measured by homeostatic model assessment 2 (mean±SEM 1.23±0.17 IPE vs 1.59±0.17 cellulose, p=0.001; 1.17±0.15 inulin vs 1.59±0.17 cellulose, p=0.009), with no differences between IPE and inulin (p=0.272). Fasting insulin was only associated positively with plasma tyrosine and negatively with plasma glycine following inulin supplementation. IPE supplementation decreased proinflammatory interleukin-8 levels compared with cellulose, while inulin had no impact on the systemic inflammatory markers studied. Inulin promoted changes in gut bacterial populations at the class level (increased Actinobacteria and decreased Clostridia) and order level (decreased Clostridiales) compared with cellulose, with small differences at the species level observed between IPE and cellulose.

**Conclusion:**

These data demonstrate a distinctive physiological impact of raising colonic propionate delivery in humans, as improvements in insulin sensitivity promoted by IPE and inulin were accompanied with different effects on the plasma metabolome, gut bacterial populations and markers of systemic inflammation.

**Significance of this study**

**What is already known on this subject?**

- Short-chain fatty acids (SCFA), derived from fermentation of dietary fibre by the gut microbiota, have been shown to improve host insulin sensitivity.
- We have previously shown that supplementing the diet with inulin-propionate ester (IPE), designed to deliver the SCFA propionate to the colon, improves glucose homeostasis in humans, but the underlying mechanisms are unclear.

**Significance of this study**

**What are the new findings?**

- Dietary supplementation with 20 g/day of IPE or the high-fermentable control fibre inulin for 42 days improved insulin sensitivity compared with the low-fermentable fibre control cellulose in adults with overweight and obesity. There were no differences with IPE compared with inulin.
- Fasting insulin following each supplementation period was associated with different plasma metabolome profiles. A positive association between plasma *N*-acetyl glycoproteins and fasting insulin was observed following cellulose supplementation, which was not found after inulin or IPE supplementation. Tyrosine (positively) and glycine (negatively) were only associated with fasting insulin following inulin supplementation.
- The improvement in metabolic health with IPE relative to cellulose supplementation was accompanied with decreased proinflammatory interleukin-8 (IL-8) levels. Analysis in vitro found that peripheral blood mononuclear cells isolated from healthy humans secrete less IL-8 in media containing sodium propionate compared with both sodium acetate and sodium chloride.
- IPE supplementation caused changes in gut bacterial populations compared with cellulose only at the species level. Inulin supplementation changed gut bacterial composition at both the class and order level, relative to cellulose, and promoted a bifidogenic effect.

**How might it impact on clinical practice in the foreseeable future?**

- Strategies that promote colonic propionate production may represent a more targeted route to improve glucose homeostasis in individual patients, depending on the underlying mechanisms contributing to the metabolic disorder.

## Introduction

In both epidemiological studies and randomised controlled trials, higher intakes of dietary fibre are associated with a reduced risk of type 2 diabetes.^1^ An improvement in metabolic health risk factors is greatest when dietary fibre intake exceeds 25 g/day,^1^ however, at the population level, average intakes are considerably below this amount.^2^ Understanding the mechanisms by which increased dietary fibre intake exerts health benefits may allow us to exploit them to prevent or treat metabolic disease.

Dietary fibre intake modulates the composition and activity of the gut microbiota.^3^ An improvement in whole-body insulin sensitivity following increased dietary fibre intake has been linked to increased colonic production of the short-chain fatty acid (SCFA) acetate, propionate and butyrate, the major end products of dietary fibre fermentation by the gut bacteria.^4 5^ Evidence in human trials has shown that increasing dietary fibre intake protects against weight gain^6 7^ and improves markers of insulin sensitivity.^8–10^ These positive effects have been observed with a number of dietary fibre supplements, including whole-grain diets,^10^ resistant starches^9^ and inulin-type fructans,^8^ which produce varying amounts of SCFAs in the gut owing to the complex interaction between the physicochemical properties of the substrate and the gut microbiota.^5 11^

SCFAs have been suggested to improve insulin sensitivity via effects on metabolic pathways and receptor-mediated mechanisms at various tissue and organ sites.^12^ Specifically, SCFAs act as ligands for G-protein-coupled receptors (GPR) free fatty acid receptor 2 (FFAR2), FFAR3 and GPR109a, which are expressed throughout the body and have been shown to modulate energy homeostasis.^13^ Our previous work has primarily focused on the role of the SCFA propionate, as a number of studies have shown that mice receiving a gut microbial transplant that promotes caecal propionate production have improved body composition and glycaemic control.^14 15^ We have described how inulin-propionate ester (IPE) can target delivery of propionate to the colon,^16 17^ and observed that long-term ingestion of 10 g/day IPE ameliorates body weight gain and the development of abdominal visceral adipose tissue in overweight human adults.^16^ A secondary outcome of this study was the observation that IPE improved glucose homeostasis, which was associated with a direct action of propionate on human islet β-cells.^18^ These studies indicated in vitro that FFAR2 is expressed in human islets and that propionate-mediated signalling potentiated glucose-stimulated insulin release and protected from apoptotic stimuli.^18^ Long-term colonic propionate delivery also reduced levels of non-esterified fatty acid (NEFA),^18^ a recognised factor that contributes to β-cell dysfunction and peripheral insulin resistance.^19^ The stimulation of FFAR2 expressed on adipocytes has previously been shown to inhibit adipocyte lipolysis and the levels of circulating NEFA.^20^

Insulin-resistant states that develop with increasing adiposity have been linked to the activation of inflammatory responses in different organ sites, including adipose tissue, liver and skeletal muscle, which increases secretion and systemic levels of proinflammatory cytokines.^21^ It is recognised that increased dietary fibre intake and SCFA production have a profound effect on inflammatory and immune function in the colon, largely through effects on the generation of regulatory T cells (Treg)^22 23^ and mucosal secretion of IgA.^24^ Previous work highlights that SCFAs can also influence inflammatory and immune responses beyond their site of production in peripheral tissues.^24–26^ Supplementing naïve T cell cultures with propionate enhanced Treg development and reduced the expansion of inflammatory Th17 cells.^26^ The improvements in glucose homeostasis we have previously observed following long-term colonic propionate delivery may be partly explained by a dampening of the low-grade systemic inflammation that accompanies obesity.

Although there is increasing evidence that gut bacteria play a role in insulin resistance, the mechanisms have not been fully elucidated. Our previous work explored the effect of increasing colonic propionate production on gut bacterial composition, using batch-culture fermentation models in vitro, and found that improvements in host metabolic health with IPE supplementation were not due to changes in the gut bacterial populations examined.^16^ However, batch-culture models lack the complexity of the human gut; 16S ribosomal RNA (rRNA) gene sequencing of stool samples would allow a more physiologically relevant and deeper interrogation of the impact of long-term colonic propionate delivery on gut bacterial composition and the association of these changes with improvements in host metabolism.

The primary aim of the present study was to elucidate the underlying mechanisms behind improvements in glucose homeostasis following long-term delivery of propionate to the human colon. In our previous studies, inulin was used as a control to account for changes to the composition and metabolic activity of the gut microbiota that may derive from the inulin content of IPE.^21^ We have previously found that 10 g/day IPE improved glucose homeostasis compared with an inulin control,^16 18^ however, inulin has itself been associated with improvements in metabolic responses when compared with a non-fermentable or low-fermentable control, particularly when supplemented in higher doses (>15 g/day).^8 27^ Consequently, the present randomised cross-over trial used 20 g/day of IPE and inulin to probe the common mechanisms underlying improvements in insulin sensitivity following dietary supplementation with a high-fermentable fibre and to differentiate them from those driven specifically from the selective delivery of propionate to the human colon with IPE.

## Methods

All participants provided informed written consent prior to the clinical trial, which was approved by the London Brent Research Ethics Committee (14/LO/0645). The study was carried out in accordance with the Declaration of Helsinki and is registered with the ISRCTN registry (ISRCTN71814178).

Men and women aged 18–65 years, with a body mass index of 25–40 kg/m^2^, were recruited. Detailed exclusion criteria are presented in the online supplementary material. The study was conducted using a randomised, double-blind, placebo-controlled, cross-over design (online supplementary figure 1). Participants received 20 g/day of a low-fermentable fibre control (cellulose; microcrystalline cellulose [ACI Group, Slough, UK]), a high-fermentable fibre control (inulin [Beneo-Orafti HP, Kreglinger Europe, Antwerpen, Belgium]) and IPE for 42 days each in a random order. The 20 g/day dose of IPE would have provided 14.6 g/day of inulin (and 5.4 g/day esterified propionate) to the diet.^16^ Cellulose was used as a negative control due to its low fermentability and consequent low SCFA production. The supplements were provided to volunteers in 10 g ready-to-use sachets and they were instructed to mix the contents into their normal diet twice a day. There was a washout period of at least 28 days between supplementation periods. The mean±SEM washout period between supplementation periods 1–2 and 2–3 was 44±6 and 44±9 days, respectively. All participants were instructed to maintain their usual dietary and physical activity habits during the study period and regular communication between participants and study investigators encouraged good compliance. Participants returned their used and unused sachets to facilitate the estimation of compliance rates.

10.1136/gutjnl-2019-318424.supp1 Supplementary data

At the end of each 42-day supplementation period, participants attended the National Institute of Health Research Imperial Clinical Research Facility to determine outcome measures. The primary outcome was change in glucose homeostasis. The day prior to the study visits, participants were requested to refrain from strenuous exercise and alcohol and to consume a standard evening meal prior to fasting overnight for >10 hours.

### Mixed meal test

A cannula was inserted into an antecubital vein and two fasting blood samples were collected >5 min apart. At 0 min, participants were served a standard liquid meal (Ensure Plus, Abbott, UK: 660 kcal; 88.9 g carbohydrate, 21.6 g fat, 27.5 g protein) that was ingested within 10 min. Postprandial blood samples were taken at 10, 20, 30, 45, 60, 90, 120 and 180 min and analysed for glucose, insulin, NEFA, active glucagon-like-peptide 1 (GLP-1), total peptide YY (PYY) and SCFA levels. ^1^H NMR spectroscopy was performed on fasting plasma samples for metabolite analysis. A detailed description of blood sample collection and analysis is presented in the online supplementary material.

### Immune and inflammatory phenotyping

IgA, IgG, IgM and C-reactive protein were measured in fasting serum samples by the Department of Chemical Pathology, Imperial College Healthcare National Health Service Trust. Interleukin (IL)-6, IL-8, IL-10, IL-12, IL-17A and tumour necrosis factor alpha (TNF-α) were measured in fasting serum using the Cytometric Bead Array (BD Biosciences, UK), according to the manufacturer’s protocol. Lipopolysaccharide binding protein (LBP) was measured in fasting serum by ELISA (Hycult Biotechnology, The Netherlands), according to the manufacturer’s protocol. Whole blood (30 mL) was collected into heparin-coated tubes and peripheral blood mononuclear cells (PBMC) were isolated using Ficoll-Hypaque (Amersham Biosciences, UK) and cryopreserved in 10% dimethyl sulfoxide/fetal calf serum. A detailed description of PBMC analysis is presented in the online supplementary material.

### Stool DNA extraction and 16S rRNA gene sequencing (metataxonomics)

A stool sample was collected from volunteers on the final day of each supplementation period. DNA was extracted from each stool sample using the PowerLyzer PowerSoil DNA Isolation Kit (Mo Bio, Carlsbad, CA, USA) following manufacturer’s instructions, with the modification that samples were beaten for 3 min at speed 8 in a Bullet Blender Storm (Chembio, St Albans, UK). A detailed description of stool sample collection and metataxonomic analysis is presented in the online supplementary material.

### Calculations and statistical analysis

A detailed description of statistical analysis is presented in the online supplementary methods. Data are presented as means±SEM and p<0.05 was considered significant.

## Results

Of 14 volunteers enrolled and randomised into the study, data were analysed from the 12 volunteers who completed all three 42-day supplementation periods. The characteristics of these volunteers at screening are presented in table 1. There was no evidence of carry-over effects in the main outcome measures across the three supplementation periods (online supplementary table 1; supplementary figure 2).

**Table 1:** Characteristics of participants at screening

Sex, n	
Male	3
Female	9
Age (years)	60±1 (49–65)
Race or ethnicity (n)	
White	11
Black	1
Weight (kg)	84.6±3.2 (68.6–113.1)
BMI (kg/m^2^)	29.8±0.9 (26.2–37.0)
HbA1c (mmol/mol)	35.5±1.0 (30–42)
Triglycerides (mmol/L)	1.0±0.1 (0.6–1.3)
Cholesterol (mmol/L)	5.2±0.3 (3.6–6.9)
LDL cholesterol (mmol/L)	3.2±0.2 (2.1–4.8)
HDL cholesterol (mmol/L)	1.5±0.1 (0.9–2.1)
Alanine Transaminase (IU/L)	22.0±2.6 (10–38)

Data are expressed as mean±SEM; ranges in parentheses.

BMI, body mass index; HDL, high-density lipoprotein; LDL, low-density lipoprotein.

**Figure 1:**
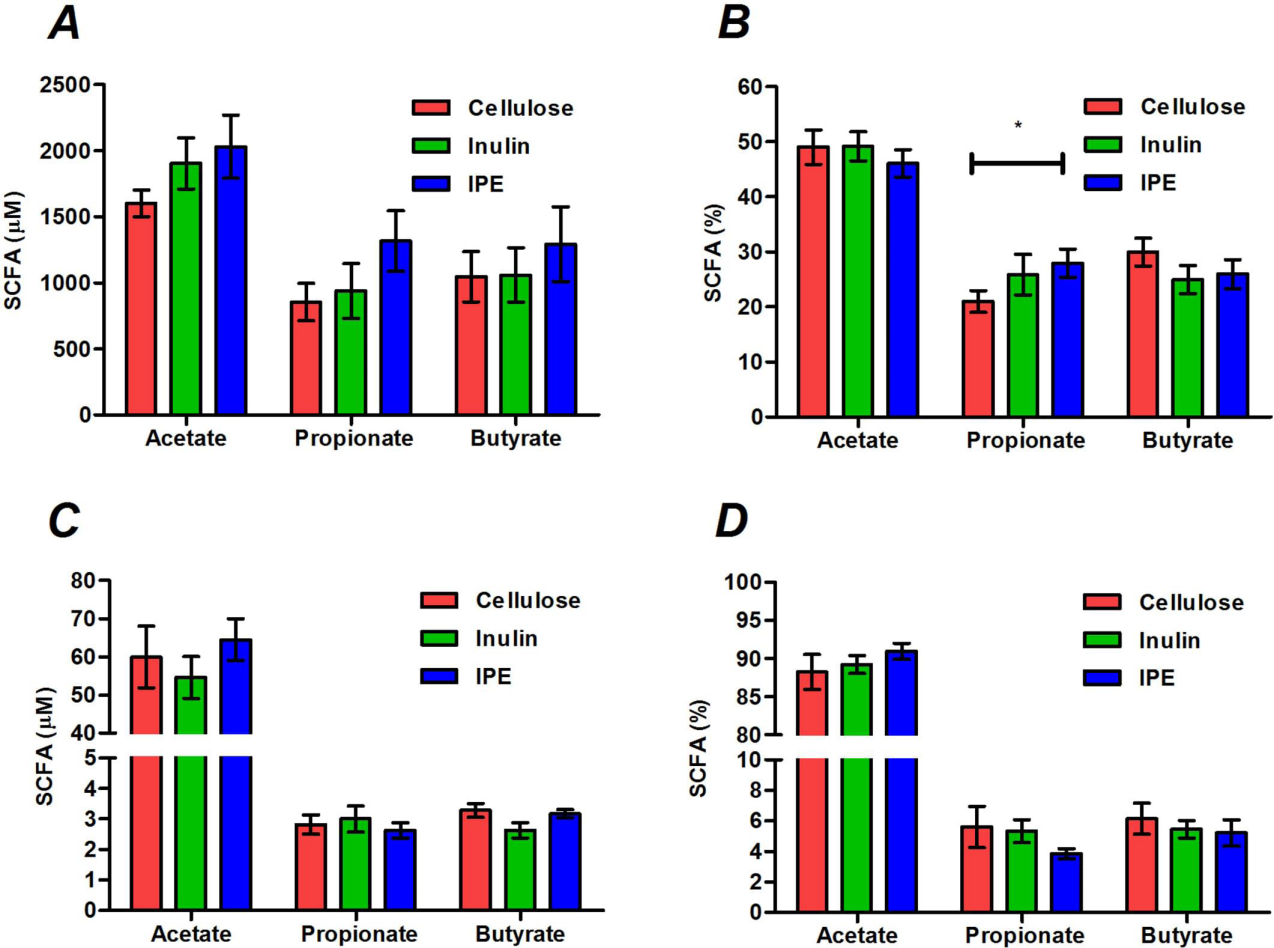
The total and molar percentages of acetate, propionate and butyrate measured in stool (A, B) and fasting serum (C, D) following 42 days of cellulose, inulin and inulin-propionate ester (IPE) supplementation. Mean±SEM (n=12). *P<0.05. Data were analysed by repeated measures analysis of variance (ANOVA) with post hoc Fisher’s least significant difference (LSD) tests. SCFA, short-chain fatty acid.

**Figure 2:**
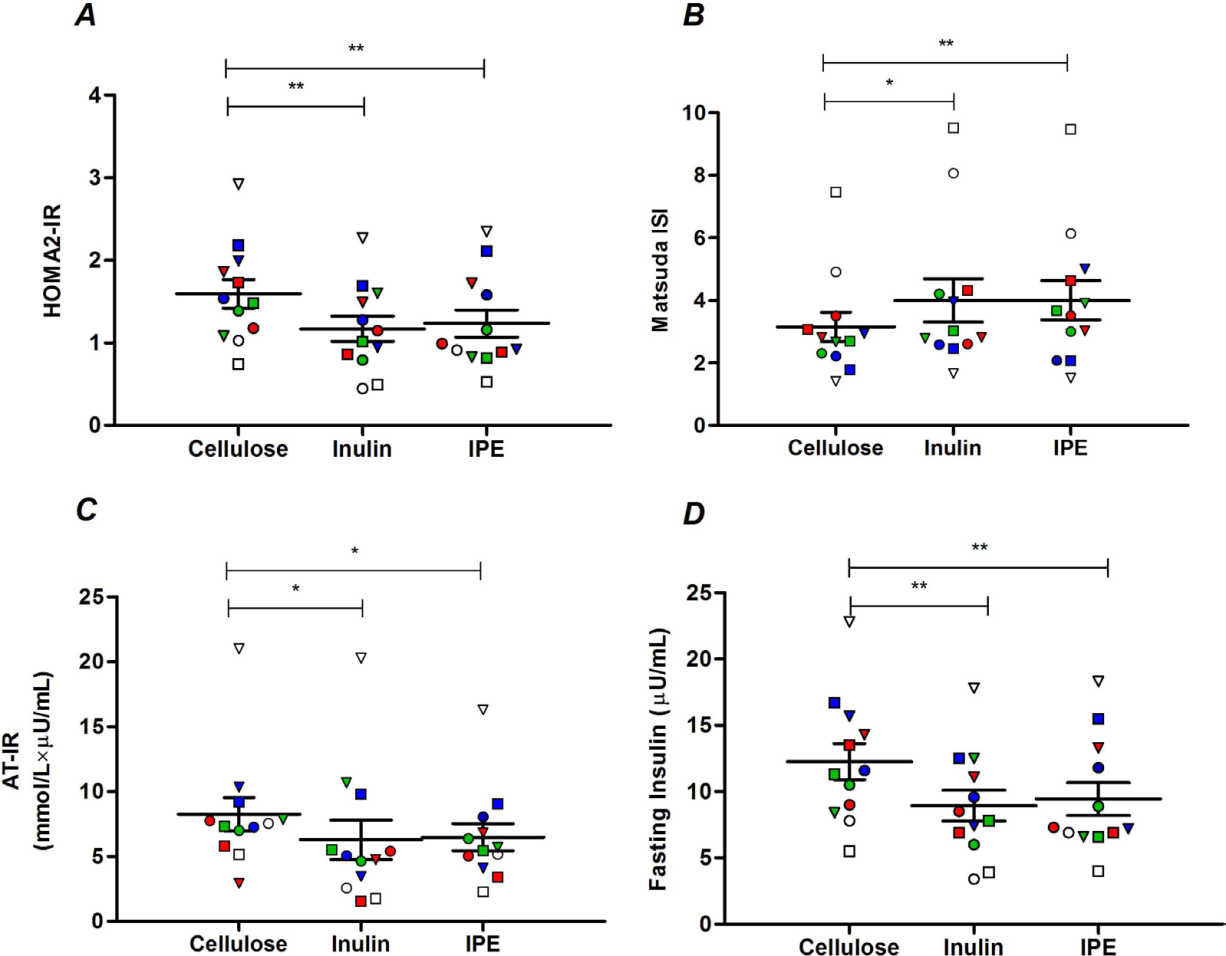
**(**A) Homeostatic model assessment 2-insulin resistance (HOMA2-IR), (B) Matsuda Insulin Sensitivity Index (ISI), (C) adipose tissue insulin resistance (AT-IR) and (D) fasting insulin following 42 days of cellulose, inulin and inulin-propionate ester (IPE) supplementation. Each individual symbol represents a volunteer and lines represent mean±SEM (n=12). *P<0.05; **P<0.01. (A) and (D) were analysed by repeated measures analysis of variance (ANOVA) with post hoc Fisher’s least significant difference (LSD) tests. (B) and (C) were analysed by Friedman test and post hoc Wilcoxon signed-rank test.

Stool concentrations of SCFAs were not different following the three supplementation periods (figure 1A), however, the molar percentage of propionate was significantly higher following IPE supplementation compared with cellulose (27.9±2.6 vs 21.0%±2.0%, p=0.019; figure 2B). There were no differences in the total or molar percentages of SCFAs in fasting or postprandial blood between supplementation periods (figure 1C,D; online supplementary table 2).

Both inulin and IPE supplementation significantly improved measures of insulin sensitivity compared with cellulose supplementation, as assessed by homeostatic model assessment 2 (1.17±0.15 inulin vs 1.59±0.17 cellulose, p=0.009; 1.23±0.17 IPE vs 1.59±0.17 cellulose, p=0.001; figure 2A) and the Matsuda Insulin Sensitivity Index (4.0±0.7 inulin vs 3.2±0.5 cellulose, p=0.014; 4.0±0.6 IPE vs 3.2±0.6 cellulose, p=0.002; figure 2B). Inulin (6.3±1.5 vs 8.3±1.3 mmol/L×µU/mL, p=0.042) and IPE supplementation (6.5±1.0 vs 8.3±1.3 mmol/L×µU/mL, p=0.042) also significantly improved adipose tissue insulin resistance compared with cellulose supplementation (figure 2C). The improvements in glucose homeostasis observed following inulin and IPE supplementation were not associated with differences in body weight, compliance, self-reported food intake, physical activity or GI side effects compared with cellulose supplementation (online supplementary tables 3 and 4).

The improvement in indices of insulin sensitivity following inulin and IPE supplementation was driven by a significant reduction in fasting insulin values compared with cellulose supplementation (9.0±1.2 inulin vs 12.3±1.4 µU/mL cellulose, p=0.004; 9.4±1.2 IPE vs 12.3±1.4 µU/mL cellulose, p=0.004; figure 2D). There were no differences in the fasting or postprandial values of other individual hormones or metabolites measured following the three supplementation periods (online supplementary table 5, supplementary figure 3). ^1^H NMR spectroscopy was performed on fasting plasma samples and the data set from the cellulose, inulin and IPE trials was modelled where fasting insulin values were used as *Y* to build an individual model per trial (table 2). This analysis identified common metabolites that were positively (valine and arginine) and negatively (high-density lipoproteins and unsaturated lipids) associated with fasting insulin following all three supplementation periods. Glutamine was negatively associated with fasting insulin following both inulin and IPE supplementation, but not cellulose, while tyrosine (positively) and glycine (negatively) were only associated with fasting insulin following inulin supplementation. *N*-acetyl glycoproteins were only positively associated with fasting insulin following cellulose supplementation.

**Table 2:** Metabolites observed in fasting plasma that were significantly associated with fasting insulin following 42 days of cellulose, inulin and inulin-propionate ester (IPE) supplementation

Trial	R^2^ Y*	Q^2^ *Y*†	Metabolite	Association‡
Cellulose	0.94	0.31	Valine	↑
			U1§	↑
			Arginine	↑
			NAC1	↑
			NAC2	↑
			HDL	↓
			Unsaturated lipids (mainly HDL)	↓
			Phosphocholine lipids	↓
Inulin	0.98	0.2	Valine	↑
			U1§	↑
			Arginine	↑
			Tyrosine	↑
			HDL	↓
			Glutamine	↓
			Unsaturated lipids (mainly HDL)	↓
			Glycine	↓
IPE	0.97	0.32	Valine	↑
			U1§	↑
			Arginine	↑
			HDL	↓
			Glutamine	↓
			Unsaturated lipids (mainly HDL)	↓
			Phosphocholine lipids	↓

*†Validation parameters of the corresponding partial least squares regression models.

‡Sign of association: ↑Upregulation and ↓downregulation at higher values of fasting insulin.

§Unknown metabolite.

HDL, high-density lipoprotein; NAC, *N*-acetyl group of glycoproteins.

**Figure 3:**
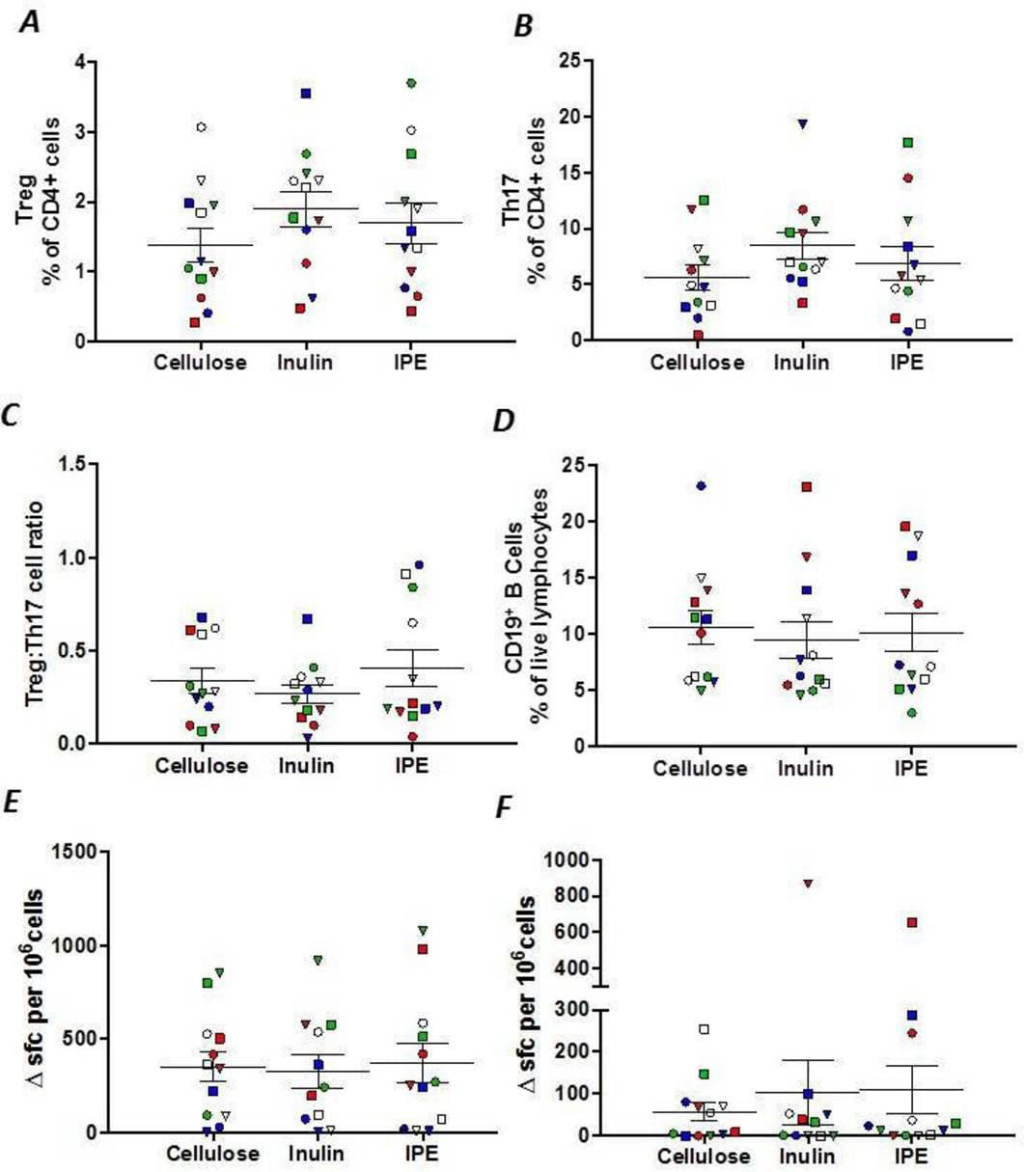
(A) The proportion of CD4^+^ Treg and (B) Th17 cells, (C) the ratio of Treg:Th17, (D) the proportion of CD19^+^ B cells, (E) interferon gamma (IFNγ) T cell spot forming cell response to the cytomegalovirus/Epstein-Barr virus/flu (CMV/EBV/flu [CEF]) peptide pool, and (F) IFNγ T cell spot forming cell response to the *Pseudomonas aeruginosa* antigen, OprF, following 42 days of cellulose, inulin and inulin-propionate ester (IPE) supplementation. Each individual symbol represents a volunteer and lines represent mean±SEM (n=12). (A) and (E) were analysed by repeated measures analysis of variance (ANOVA). (B), (C), (D) and (F) were analysed by Friedman test. sfc, spot forming cell.

PBMCs obtained from participants following the three supplementation periods were stained with an immune-phenotyping antibody panel (online supplementary table 6) for multiparameter flow cytometry to investigate potential modulation of lymphocyte subsets (figure 3A–F). The mean proportion of Treg among CD4^+^ T cells in the periphery was increased with inulin and IPE supplementation compared with cellulose, although this did not reach significance (p=0.104; figure 3A). In addition, there were no differences in the proportion of peripheral Th17 cells (p=0.179; figure 3B), the ratio of Treg:Th17 cells (p=0.758; figure 3C) or proportion of CD19^+^ B cells (figure 3D, p=0.920) between supplementation periods. Given interest in the potential of SCFA supplementation to modulate T cell function, we also examined T cell effector recall responses to antigens using the cytomegalovirus/Epstein-Barr virus/flu (CEF) viral peptide pool and a recombinant *Pseudomonas* antigen, OprF. There were no differences in T cell response to CEF or OprF stimulation between supplementation periods (figure 3E,F).

**Figure 4:**
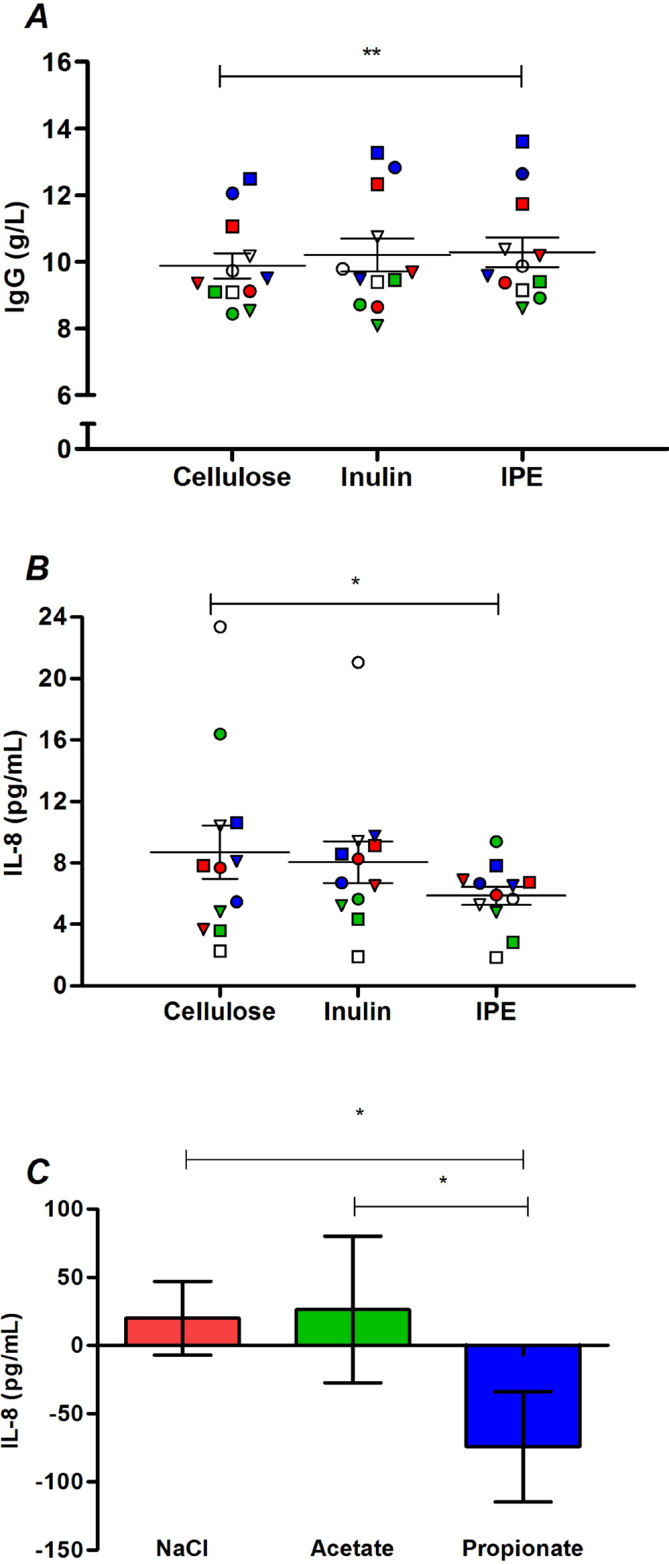
(A) IgG and (B) IL-8 in fasting serum following 42 days of cellulose, inulin and inulin-propionate ester (IPE) supplementation. Each individual symbol represents a volunteer and lines represent mean±SEM (n=12). (C) IL-8 release from peripheral blood mononuclear cells (PBMC) isolated from 12 healthy volunteers cultured for 48 with 4 mM sodium chloride, 4 mM sodium acetate and 4 mM sodium propionate. The concentration of IL-8 produced following culture with media alone was subtracted from the treated samples to determine change in IL-8. Mean±SEM (n=12). *P<0.05; **P<0.01. (A) was analysed by repeated measures analysis of variance (ANOVA) with post hoc Fisher’s least significant difference (LSD) tests. (B) and (C) were analysed by Friedman test and post hoc Wilcoxon signed-rank test. IL-8, interleukin-8.

Differences in inflammatory and immune markers following the three supplementation periods are presented in online supplementary table 7. IL-17 and TNF-α are not shown, as only three volunteers had detectable values for these analytes. IPE supplementation significantly increased IgG levels compared with cellulose supplementation (10.29±0.45 vs 9.89±0.38 g/L, p=0.002; figure 4A). In addition, IPE supplementation significantly decreased IL-8 levels (figure 4B) compared with cellulose supplementation (5.86±0.59 vs 8.69±1.74 pg/mL, p=0.041), with a trend observed for a difference between IPE and inulin supplementation values (5.86±0.59 vs 8.05±1.36 pg/mL; p=0.050). Analysis in vitro (figure 4C) observed that healthy human PBMCs cultured with sodium propionate secrete significantly less IL-8 compared with both sodium chloride (p=0.021) and sodium acetate (p=0.040).

Both IPE and inulin supplementation periods decreased the diversity of bacterial species compared with cellulose (online supplementary figure 4A–C). The decrease in bacterial diversity observed with both inulin and IPE was not related to differences in richness, while there was a decreased enrichment (changes in evenness) with inulin compared with cellulose supplementation. We found no differences between supplementation periods on gut bacterial populations at the phylum level. At the class level, we observed an increase in Actinobacteria and decrease in Clostridia with inulin supplementation compared with cellulose (online supplementary figure 5A), while at the order level we found a decrease in the proportion of Clostridiales with inulin supplementation compared with cellulose (online supplementary figure 5B). At the species level, we found that the supplementation with inulin resulted in a higher proportion of *Anaerostipes hadrus*, *Bifidobacterium faecale* and *Bacteroides caccae* and a lower proportion of *Blautia obeum, Blautia luti*, *Oscillibacter* spp, *Blautia faecis* and *Ruminococcus faecis,* compared with cellulose (online supplementary figure 6). IPE supplementation resulted in a higher proportion of *Bacteroides uniformis* and *Bacteroides xylanisolvens* and a lower proportion of *B. obeum* and *Eubacterium ruminantium* compared with cellulose (online supplementary figure 7). We also found that the IPE supplementation resulted in a higher proportion of *Fusicatenibacter saccharivorans* and a lower proportion of *A. hadrus, B. faecale* and *Prevotella copri* compared with the inulin supplementation period (online supplementary figure 8).

## Discussion

The aim of the present study was to explore the mechanisms behind changes in insulin sensitivity observed when selectively increasing propionate delivery to the human colon compared with a high-fermentable fibre. We hypothesised that supplementing the diet of adults with overweight and obesity with 20 g IPE for 42 days would improve insulin sensitivity compared with both a high-fermentable (inulin) and low-fermentable (cellulose) fibre control through modulatory effects on gut bacterial composition, reductions in NEFA levels and improvements in inflammatory markers. In our previous work, we observed that dietary supplementation with 10 g/day IPE improved glucose homeostasis compared with an inulin control.^16 18^ In the present study, we found that dietary supplementation with 20 g/day IPE promoted no superior impacts on measures of glucose homeostasis compared with inulin, yet both IPE and inulin improved insulin resistance relative to cellulose.

The improvements in insulin sensitivity observed with IPE or inulin appear to encompass separate effects on gut bacterial communities and markers of systemic inflammation. Furthermore, we modelled fasting insulin responses with plasma metabolome profiles and observed that different metabolites were associated with fasting insulin after each supplementation period. We observed a positive association between plasma *N*-acetyl glycoproteins and fasting insulin after cellulose supplementation, which was not found following inulin or IPE supplementation. *N*-acetyl glycoproteins have previously been linked with increased insulin resistance^28^ and elevated risk of type 2 diabetes.^29^ Following both inulin and IPE supplementation, fasting insulin was negatively associated with glutamine. Previous studies have observed a similar inverse relationship between glutamine and insulin resistance.^30^ Tyrosine and glycine have also been identified as biomarkers of glucose homeostasis^31 32^ and we found that fasting insulin was only associated with these amino acids following inulin supplementation. Our data therefore indicate that the observed improvement in insulin sensitivity following inulin supplementation was related to a favourable modulation of amino acid metabolism.

Previous reports have linked raised colonic SCFA production with anti-inflammatory responses, thus we explored the effects of IPE supplementation on a range of systemic inflammatory and immune parameters. Findings from murine models would predict that raising colonic propionate delivery would expand the proportion of Treg cells, while proinflammatory Th17 cells would be lowered,^23 26 33^ although the majority of effects have been limited to colonic immune subsets rather than systemic populations. Until the present study, there has been limited opportunity to explore these impacts in a human cohort. Both inulin and IPE supplementation induced a modest enhancement in peripheral Tregs, although this did not reach significance. Neither IPE nor inulin supplementation had significant effect on T cell effector recall responses to antigens. We found that IPE significantly decreased circulating IL-8 levels compared with cellulose supplementation. This outcome was supported by our observation that propionate significantly reduced the secretion of IL-8 from cultured human PBMCs. SCFAs have recently been shown to supress IL-8 production from human intestinal Caco-2 cells^34^ and human umbilical vein endothelial cells.^35^ It was also shown that propionate had a greater potency to suppress IL-8 expression compared with acetate^34^ and the present study observed that the reduction in IL-8 from human PBMCs was only found with propionate and not acetate. IL-8 is a key chemokine bringing neutrophils and macrophages to sites of inflammation and contributing to the proinflammatory profile characteristic of metabolic syndrome.^21^ This chemotaxis can subsequently impact processes that impair insulin signalling,^21^ and studies have associated elevated levels of IL-8 with increased insulin resistance in humans.^36 37^ Consequently, the inhibition of IL-8 by IPE supplementation appears to be driven by the selective delivery of propionate to the colon and may partly explain the observed improvements in glucose homeostasis.

IPE supplementation also promoted a small but highly significantly increase in serum IgG levels relative to cellulose. The physiological relevance of this minor elevation in peripheral IgG in humans is unclear, but is consistent with an observation from rodent studies that SCFA treatment increases the expression of genes that enhance antibody production in B cells, which improves systemic immune responses.^24^ To the best of our knowledge, this is the first evidence in vivo in humans that selectively increasing colonic propionate delivery increases circulating IgG levels and it would be of future interest to determine if this effect of IPE on adaptive immunity can decrease susceptibility to pathogen exposure.

Metataxonomic analysis of stool samples compared the impact of each supplementation period on gut bacterial composition. The improvements in metabolic markers following inulin and IPE supplementation were observed despite decreased stool bacterial diversity compared with cellulose. This outcome appears counterintuitive given the commonly accepted association in humans between a lower gut bacterial diversity and poor health.^38^ Nevertheless, previous studies have also found that dietary supplementation of a single fermentable substrate can reduce indices of stool bacterial diversity in humans,^39^ yet improve metabolic responses.^40^ No changes were found at the phylum level and differences at the class and order level were only detected between the inulin and cellulose supplementation periods. This finding supports the concept that dietary intervention in ‘free-living’ humans has a more selective effect on gut bacterial species compared with the extensive shift in gut bacterial populations reported in rodent models.^27 41^ We observed that both IPE and inulin decreased the abundance of selective species of Firmicutes (IPE: *B. obeum*, *E. ruminantium*; inulin: *B. obeum*, *B. luti*, *B. faecis*, *R. faecis*, *Oscillibacter* spp) and stimulated the growth of *Bacteroides* spp (IPE: *B. uniformis*, *B. xylanisolvens*; inulin: *B. caccae*) compared with cellulose. This observation supports our previous work in vitro showing that both IPE and inulin increase the abundance of *Bacteroides* spp.^16^ The improvement in metabolic homeostasis following dietary supplementation with inulin-type fructans has been linked to an increased growth of *Bifidobacterium* spp,^27 42^ which have also been shown to decrease in obesity and type 2 diabetes.^43 44^ Interestingly, only inulin supplementation promoted a bifidogenic effect, with increased abundance of *B. faecale* compared with both cellulose and IPE. The difference between inulin and IPE confirms our observations in vitro^16^, but was unanticipated considering that 20 g/day IPE supplementation would itself provide 14.6 g/day of inulin to the diet, and previous work has demonstrated that lower intakes (12 g/day) stimulate *Bifidobacterium* abundance in the human faecal microbiota.^41^ We conclude that the high levels of propionate delivered to the colon by IPE inhibit the bifidogenic action of inulin fermentation. Previous investigations in both animal and human models have linked the increased abundance of bifidobacteria from feeding inulin-type fructans with an improved gut barrier function.^27 42^This change has been associated with improvements in host glucose homeostasis through reduced endotoxaemia, which can directly induce insulin resistance in peripheral tissues.^45^ It is therefore surprising that the observed changes in gut bacterial populations with inulin supplementation were not associated with altered inflammatory and immune responses. Inflammatory changes were, however, only measured in peripheral blood samples and we cannot exclude the possibility that the changes in gut bacterial composition promoted by inulin supplementation had localised effects on inflammatory responses within the intestinal mucosal environment.^46 47^ Despite the increase in bifidobacteria following inulin supplementation there were no differences in circulating LBP following the three supplementation periods. The bifidogenic action of inulin fermentation and reduced endotoxaemia reported in rodent studies has commonly been found in animals fed a high-fat diet, which promotes considerable impairments to gut integrity.^42^ In comparison, all volunteers in the present study had relatively low levels of LBP (<15 µg/mL), suggesting there was limited scope for the bifidogenic effect of inulin to translate into improvements in gut permeability.

We hypothesised that IPE supplementation would reduce NEFA levels, as found in our previous experiments,^18^ yet fasting and postprandial NEFA concentrations were unaffected after IPE feeding relative to both inulin and cellulose. The major methodological differences that may explain this discrepancy are the shorter supplementation period in the present study and the fact that the IPE was not provided with the standard test meal. This methodological detail may also explain why we were unable to detect any differences in circulating SCFAs or anorectic hormone release between the three supplementation periods. Blood samples were collected and analysed following an overnight fast and up to 180 min following a standard mixed meal test. A longer study protocol (>6 hours) with the addition of the fermentable substrates to the test meal may have been necessary to observe differences in peripheral circulating levels of SCFAs and their potential effects on intestinal PYY and GLP-1 release.^48^ We chose not to add the fibre supplements to the test meal so that any observed effect on postprandial metabolism was independent of possible acute alterations in digestion and absorption caused by the physiochemical properties of each fibre supplement.

In summary, in a cohort of adults with overweight and obesity both inulin and IPE supplementation improved measures of insulin resistance relative to cellulose, however, there was no significant difference between IPE and inulin. Despite this comparable improvement to metabolic health, IPE supplementation generated distinct effects on gut bacterial species and markers of systemic inflammation and immune function compared with those observed with the supplementation of inulin alone. Taken together, the present study suggests that manipulating the colonic fermentation profile of a dietary fibre in favour of propionate promotes selective effects on the mechanisms that contribute to metabolic dysregulation. It would be of interest to establish the individual effects of delivering acetate and butyrate to the colon as, in the future, this would support the development of fermentable carbohydrate that delivers a specific SCFA profile to improve metabolic health and glucose homeostasis.
