# Supplementary material for: Dietary supplementation with inulin-propionate ester or inulin improves insulin sensitivity in adults with overweight and obesity with distinct effects on the gut microbiota, plasma metabolome and systemic inflammatory responses: a randomised cross-over trial
Source: Gut. 2019 Apr 10;68(8):1430–8. doi: 10.1136/gutjnl-2019-318424 (PMC6691855; doi:10.1136/gutjnl-2019-318424)
Supplement: Supplementary data [file gutjnl-2019-318424supp001.pdf]

SUPPLEMENTARY MATERIAL

SUPPLEMENTARY METHODS

Potential participants were excluded if they met any of the following criteria: clinically significant illness (including type 1 or type 2 diabetes), history of gastrointestinal disease (including inflammatory bowel disease or irritable bowel syndrome), medication known to affect body weight or glucose homeostasis, anti-inflammatory medication, a weight loss of 3 kg or greater in the preceding two months, smoking, substance abuse, psychiatric illness, and any abnormalities detected on physical examination, electrocardiography, or screening blood tests (measurement of complete blood count, electrolytes, glycosylated haemoglobin (HbA1c), thyroid function and liver function). Participants were ineligible if they had taken antibiotics in the previous 3 months and were excluded if they took antibiotics during the study period. Women were ineligible if they were pregnant or breast-feeding.

The randomisation procedure was conducted by an investigator not directly involved in the study with the use of opaque, sealed, sequentially numbered envelopes that each contained a random order that volunteers would receive the three supplements. The envelopes were stored securely and opened in sequence by an investigator (ESC) as each participant was enrolled.

Body weight and composition

Body weight, fat mass (FM) and fat free mass (FFM) were measured with bioelectrical impedance (Tanita BC-418MA, Japan). Participants were asked to change into lightweight hospital scrubs and to void their bladder before measurements were taken.

Mixed meal test (MMT)

Plasma glucose was measured from blood collected into sodium fluoride plasma tubes and assayed using an Abbott Architect ci8200 analyser (Abbott Diagnostics, USA). Insulin-like immunoreactivity was measured from blood collected into serum-separating tubes and quantified using an ultra-sensitive human insulin radioimmunoassay (RIA) (Millipore, USA). Serum short chain fatty acids (SCFAs) were measured with the use of an Agilent 7000C Triple Quadrupole GC/MS System according to a previously published method [1]. NEFA were measured using an ILAB 650 Clinical Chemistry Analyzer (Instrumentation Laboratory, UK). 2 mL blood was collected into heparin-coated tubes containing 40μL of Aprotinin (Bayer, UK) and total peptide YY

(PYY) and active glucagon-like-peptide 1 (GLP-1) were measured in the same assay using a Milliplex MAP kit (Millipore, USA).

Sample preparation for nuclear magnetic resonance (NMR) analysis

The plasma samples were thawed, vortexed and allowed to stand for 10 min at room temperature. The samples were centrifuged at 18 000 g at 4 ºC for 10 min. 300 µL of supernatant was mixed with 300 µL of 75 mM Na_2HPO_4 buffer (pH 7.4, LC-MS water/D_2O (80:20)), 2 mM sodium azide and 0.08% w/v of TSP[2]**.** One quality control (QC) sample was analysed every ten samples.

^{1}H NMR spectroscopy

Water-suppressed ^{1}H NMR spectroscopy was performed at 310 K on a Bruker 600 MHz Avance III HD spectrometer equipped with a 5-mm BBI Z-gradient probe and automated tuning and matching (Bruker Biospin, Karlsruhe, Germany). The ^{1}H NMR spectra were acquired using standard one-dimensional pulse sequence, with saturation of the water resonance (noesygppr1d, standard Bruker pulse program) during both the relaxation delay (RD = 4s) and mixing time (τ_m = 10ms). The two magnetic field z-gradients implemented by this pulse sequence are applied for 1 ms. The receiver gain was set to 90.5 and acquisition time (ACQ) to 2.73s for all experiments. Each ^{1}H NMR spectrum was acquired using 4 dummy scans, 32 scans, 64K time domain points and with a spectral window of 20 ppm. Following the recording of the 1D NOESY-presat, one-dimensional CPMG with water saturation was acquired using the Carr–Purcell–Meiboom–Gill pulse sequence (cpmgpr1d). The acquisition parameters are set up in the same way as the 1D NOESY-presat, with the addition of the spin-echo delay (Δ1/Δ2) that is set at 0.3 ms and the implementation of 128 loops for T_2 filter (L4). Prior to Fourier transformation of one-dimensional experiments, free induction decays were multiplied by an exponential function corresponding to a line broadening of 0.3 Hz.

^{1}H–^{1}H 2D J-resolved experiments were acquired to detect the J-couplings in the second dimension, with suppression of the water resonance (jresgpprqf). The parameters used for this pulse sequence were as follows: 16 dummy scans and 2 scans, 8K points with spectral window of 16.7 ppm for f2 and 40 increments with spectral window of 78 Hz for f1.Incremented delay of 3µs, RD of 2 s, and ACQ of 0.41 s. The receiver gain was set to 90.5. A sine-bell apodization function was applied on both dimensions, followed by Fourier transformation, tilting by 45°, and symmetrization along f1.

Selective 1D TOCSY sequence and 2D NMR experiments with water pre-saturation during relaxation delay including gradient ^{1}H–^{1}H COrrelation SpectroscopY 45º (gCOSY-45) and ^{1}H–^{13}C Heteronuclear Single Quantum Coherence (HSQC) were acquired for representative samples to aid

the spectral assignment. Only the selective 1D TOCSY experiments were acquired on a Bruker 800 MHz spectrometer equipped with a 5 mm CPTCI Z-gradient cryoprobe, and automated tuning and matching.

NMR data pre-processing

The multivariate data analysis was performed on the 1D ^{1}H CPMG spectra. Each spectrum (~19K spectral variables) was automatically phased and baseline corrected and then digitized over the range δ -0.5 to 11 and imported into MATLAB (2014a, MathWorks, Natick, U.S.A.). The spectral regions corresponding to the internal standard (δ -0.5 to 0.5), water (δ 4.7 to 5.5) and noise (δ 9.3 to 11.00) were excluded. The spectra of plasma were referenced to the doublet of the anomeric proton signal of α-glucose at δ 5.23 ppm. The spectral regions occupied by the internal standard (δ -0.5 to 0.7), water (δ 4.3 to 5.1) and noise (δ 8.5 to 11.00) were excluded. Prior to multivariate data analysis, the spectra from both data sets were normalized using the probabilistic quotient method (PQN)[3].

NMR statistical analysis

The data sets (Cellulose, inulin and IPE) were modelled separately using Partial Least Squares (PLS) regression in a Monte-Carlo cross-validation (MCCV) framework[4 5], whereby fasting insulin from the corresponding trial were used as Y to build an individual model per supplementation period.

Under MCCV approach the NMR data set is split into many different training and test sets, and each training and test set is auto-scaled using training set parameters (mean, standard deviation), to obtain a robust and reliable estimation of the regression coefficients and predicted scores[4]. Specifically, for each of 100 models, 1/5th of the data is left out and the variability of the regression coefficients is calculated by running an additional 25 models with bootstrap resampling from the training data[5]. The 100 models give a robust estimate of the mean regression coefficients and the 2,500 bootstrap models are used to estimate the variance[4 5]. Using the variance and mean regression coefficient, a t-score for each variable and subsequently *P*-value were calculated. *P*-values are then adjusted for multiple testing by calculating the False Discovery Rate (FDR) using Benjamini-Hochberg method. The goodness of fit (R^2Y) of the MCCV models was calculated across all models using the training data and the goodness of prediction (Q^2Y) for the test data.

The variable significance was represented as a “Skyline” (S_i) value:

$$\boldsymbol{S_i = -log_{10}(pFDR)} \times (\boldsymbol{\beta_i}/|\boldsymbol{\beta_i}|)$$

Where, β_i is the ith regression coefficient and *p*FDR the *P*-value after Benjamini-Hochberg correction for multiple testing. Variables with *p*FDR < 0.05 are considered to be significant in the MCCV framework.

Identification of Metabolites.

Subset optimization by reference matching (STORM) was used to identify metabolites using the correlation structure of 1D ^1H NMR data[6]. Internal and external databases such as the Human Metabolome Data Base (HMDB; http://hmdb.ca/) or the Biological Magnetic Resonance Data Bank (BMRB; http://www.bmrb.wisc.edu) were used for confirmation of assignments.

Immune and inflammatory phenotyping

Cell surface phenotype of thawed PBMCs was analysed by multi-parameter flow cytometry on a BD FACS Aria II, using the antibody panel indicated in Suppl. Table 7. T cell responder status of donor PBMC was tested in an IFN□ ELIspot assay of recall responses to recombinant outer membrane porin F (OprF) antigen (at a final concentration of 25µg/ml) of *Pseudomonas aeruginosa* [7], and to the 'CEF peptide pool' of EBV/Flu/CMV epitopes (Mabtech) at the same concentration. IFNγ ELISpot assays used reagents from Mabtech (Stockholm, Sweden). Two x 10^5 PBMC were incubated in pre-coated IFNγ ELISpot plates, in triplicate, with 25 µg/ml of the indicated antigens, in RPMI-1640 medium containing 2 mM L-glutamine, 50U/ml penicillin G, 50µg/ml streptomycin sulphate, 10% FCS. Cells were cultured at 37° in 5% CO_2 for 24h before assay development using HRP-labelled detection antibody and Tetramethylbenzidine (TMB) substrate. Plates were dried and then counted using an AID ELISpot reader (Autoimmun Diagnostika GMBH, Straßberg, Germany). The choice of OprF as a model recall antigen was based on our earlier observation that the majority of individuals display this T cell response[7].

The effect of sodium acetate and sodium propionate on IL-8 release from human PBMCs

PBMCs were freshly isolated from 12 healthy volunteers and cultured with media alone (RPMI containing 10% fetal calf serum, 2mM L-Glutamine, 50 U/ml Penicillin G and 50 µg/ml Streptomycin sulphate), or with media containing 4mM sodium propionate, 4mM sodium acetate or 4mM sodium chloride. Supernatants were collected after 48 hours of culture and the amount of IL-8 in each supernatant measured by ELISA (Bio-Techne, USA). The concentration of IL-8 produced following culture with media alone was subtracted from the treated samples to determine change in IL-8.

Stool collection, DNA extraction and 16S rRNA gene sequencing (Metataxonomics)

Participants were provided with a standardised kit and instructions for the collection of stool samples, which were all collected in the home environment on the day prior to study visits. Participants were provided with disposable sterile towel (Dynarex, USA) to line the toilet bowl for stool collection. A sample of stool was then immediately transferred to universal 30ml stool collection container and frozen at -20^{0}C. On the study morning the stool samples were transferred using an insulated freezer bag (Kingscroft Logistics Ltd, UK) and frozen ice packs to prevent the sample thawing. Upon arrival at the NIHR Imperial Clinical Research Facility the samples were transferred to -80^{0}C until analysis. DNA was extracted from approximately 250 mg of stool and stored at -80°C until ready to use. Stool samples were stored for between 3-13 months before being processed and analysed in a single batch.

Sample libraries were prepared following Illumina's 16S Metagenomic Sequencing Library Preparation Protocol with the following modifications. First, the index PCR reactions were cleaned up and normalised using the SequalPrep Normalization Plate Kit (Life Technologies, Paisley, UK). In addition, sample libraries were quantified using the NEBNext Library Quant Kit for Illumina (New England Biolabs, Hitchin, UK).

Sequencing was performed on an Illumina MiSeq platform (Illumina Inc., Saffron Walden, UK) using the MiSeq Reagent Kit v3 (Illumina) using paired-end 300bp chemistry. The resulting data was analysed using the Mothur v.1.35.1 package following the MiSeq SOP Pipeline[8]. The Silva bacterial database was used for sequence alignments (www.arb-silva.de/) and the RDP database reference sequence files were used for classification of sequences using the Wang method[9]. The Wilcoxon signed-rank test was used to compare the effects of the different dietary interventions on the stool bacterial communities.

Stool samples were analysed for SCFAs according to a previously published method [10].

Self-reported food intake, physical activity and gastrointestinal adverse events

Energy and macronutrient intake was recorded with food diaries during the final 3 days of each supplementation period (Dietplan 6.0; Forestfield Software Ltd, UK). Physical activity was assessed during the final 7 days of each supplementation period using the short self-administered format of the International Physical Activity Questionnaire (IPAQ)[11]. Participants were asked to complete 100 mm visual analogue scales (VAS) to assess the occurrence of gastrointestinal adverse effects experienced during each 42 day supplementation period[12].

Calculations and statistical analysis

Data from Robertson and colleagues[13] were used to estimate the required sample size. A power calculation confirmed that 12 participants would be sufficient to detect a 0.50 unit change in insulin sensitivity, assessed by homeostatic model assessment (HOMA-IR)[14], with a standard deviation (SD) of 0.47 unit (α=0.05, power=0.90). Time course data from the MMT were analysed by calculating areas under the curve (AUC) using the trapezoid rule. Insulin sensitivity was assessed by homeostatic model assessment 2 – insulin resistance (HOMA2-IR) and the Matsuda Insulin Sensitivity Index (ISI)[15 16]. Adipose tissue insulin resistance (AT-IR) was calculated using the product of fasting insulin and NEFA levels[17]. An oral disposition index, which provides a measure of β-cell function adjusted for insulin sensitivity, was calculated as $AUC_{Insulin/Glucose}$ divided by HOMA2-IR[18]. All statistical analyses were carried out with SPSS version 23.0 for Windows (SPSS Inc, USA). Repeated measures ANOVA with post hoc Fishers LSD tests were used for the comparison of variables with a single measurement following each supplementation period. Data were checked for normality using the Shapiro-Wilk Test. Non-parametric analyses (Friedman test and *post hoc* Wilcoxon signed rank test) were performed on data that were not normally distributed.

SUPPLEMENTARY TABLES

Suppl. Table 1. The effect of supplementation order on primary outcome measures.

	Supplementation Period 1	Supplementation Period 2	Supplementation Period 3	P Value
**Fasting Glucose (mmol/L)**	**5.3±0.2**	**5.3±0.1**	**5.1±0.1**	**0.238**
**Postprandial Glucose (mmol/L×min)**	**1149±60**	**1182±68**	**1164±53**	**0.597**
**Fasting Insulin (µU/mL)**	**9.9±1.3**	**9.9±1.2**	**10.8±1.5**	**0.592**
**Postprandial Insulin (µU/mL×min)**	**19075±2411**	**17801±2150**	**18230±2202**	**0.632**
HOMA2-IR	**1.3±0.2**	**1.3±0.2**	**1.4±0.2**	**0.664**
Matsuda ISI	**67±11**	**67±11**	**67±12**	**0.920†**
AT-IR (mmol/L× µU/mL)	**6.1±1.1**	**7.7±1.4**	**7.2±1.4**	**0.174†**
**IgA (g/L)**	**2.59±0.29**	**2.54±0.26**	**2.54±0.25**	**0.501**
**IgG (g/L)**	**10.13±0.48**	**10.19±0.45**	**10.06±0.41**	**0.779†**
**IgM (g/L)**	**0.82±0.11**	**0.82±0.10**	**0.80±0.10**	**0.924†**
**C Reactive Protein (mg/L)**	**1.66±0.37**	**1.81±0.48**	**2.31±0.69**	**0.856†**
**IL-6 (pg/mL)**	**2.62±0.82**	**1.95±0.40**	**2.12±0.74**	**0.368†**
**IL-8 (pg/mL)**	**7.92±1.62**	**7.81±1.53**	**6.86±0.59**	**0.558†**
**IL-10 (pg/mL)**	**0.30±0.08**	**0.29±0.08**	**0.29±0.07**	**0.144†**
**IL-12 (pg/mL)**	**21.08±3.33**	**22.50±2.88**	**25.65±3.90**	**0.432**
**LBP (ng/mL)**	**11.97±0.86**	**11.42±0.79**	**11.65±0.76**	**0.751**

Postprandial values calculated as AUC. Data are expressed as mean ± SEM (n=12). P value is from repeated measures ANOVA. †P value from Friedman test.

Suppl. Table 2. The total and molar percentages of acetate, propionate and butyrate measured in stool and peripheral serum following 42 days of cellulose, inulin and inulin propionate ester (IPE) supplementation.

	Cellulose	Inulin	IPE	P Value
Stool				
**Acetate (µmol/L)**	**1600±101**	**1902±193**	**2030±238**	**0.289**
**Propionate (µmol/L)**	**756±126**	**1075±199**	**1316±288**	**0.161**
**Butyrate (µmol/L)**	**1084±179**	**1073±201**	**1292±282**	**0.729**
**Acetate (%)**	**49±3**	**49±3**	**46±3**	**0.570**
**Propionate (%)**	**21±2**	**26±4**	**28±3^**	**0.044**
**Butyrate (%)**	**30±3**	**25±3**	**26±3**	**0.248**
Fasting Serum				
**Acetate (µmol/L)**	**60.0±8.1**	**54.6±5.5**	**64.6±5.5**	**0.567**
**Propionate (µmol/L)**	**2.8±0.3**	**3.0±0.4**	**2.6±0.3**	**0.794**
**Butyrate (µmol/L)**	**3.3±0.2**	**3.0±0.2**	**3.2±0.1**	**0.737**
**Acetate (%)**	**88±2**	**89±1**	**91±1**	**0.445**
**Propionate (%)**	**6±1**	**5±1**	**4±1**	**0.368**
**Butyrate (%)**	**6±1**	**5±1**	**5±1**	**0.080**
Postprandial Serum				
**Acetate (µmol/L)**	**7251±406**	**7276±458**	**8351±527**	**0.154**
**Propionate (µmol/L)**	**445±55**	**482±36**	**459±34**	**0.847**
**Butyrate (µmol/L)**	**486±26**	**484±25**	**498±16**	**0.800**
**Acetate (%)**	**88±1**	**88±1**	**89±1**	**0.567**
**Propionate (%)**	**5±1**	**6±1**	**5±1**	**0.794**
**Butyrate (%)**	**6±1**	**6±1**	**6±1**	**0.495**

Data are expressed as mean ± SEM (n=12). ^=$p<0.05$, cellulose vs. IPE. P value is from repeated measures ANOVA.

Suppl. Table 3. Body weight and body composition following 42 days of cellulose, inulin and inulin propionate ester (IPE) supplementation.

	Cellulose	Inulin	IPE	P Value
**Body Weight (kg)**	**85.3±3.2**	**85.1±3.2**	**84.9±3.2**	**0.742**
**BMI (kg/m2)**	**30.0±0.9**	**30.0±0.9**	**29.9±0.9**	**0.773**
**Body Fat (%)**	**37.5±2.1**	**37.8±2.1**	**37.4±2.0**	**0.083†**
**Body Fat (kg)**	**31.8±1.9**	**32.0±1.9**	**31.6±1.9**	**0.718**
**FFM (kg)**	**53.5±3.2**	**53.1±3.1**	**53.3±3.1**	**0.915†**

Data are expressed as mean ± SEM (n=12). P value is from repeated measures ANOVA. †P value from Friedman test.

Suppl. Table 4. Self-reported food intake, compliance, physical activity and gastrointestinal side-effects during 42 days of cellulose, inulin and inulin propionate ester (IPE) supplementation.

	Cellulose	Inulin	IPE	P Value
Energy (kcal/day)	2106±119	1919±110	2025±185	0.311
Protein (g/day)	90±6	83±6	86±7	0.340
Carbohydrate (g/day)	228±18	202±14	205±18	0.258
Fat (g/day)	84±7	77±6	89±11	0.245
Fibre (g/day)	12±1	10±2	11±1	0.568
Alcohol (g/day)	15±4	16±5	13±6	0.814
Total Physical Activity (MET-h/week)	82±42	55±15	57±14	0.862†
Compliance (%)	95±2	96±1	93±2	0.739†
Stomach Discomfort (mm)	11±6	17±6	18±9	0.518†
Nausea (mm)	1±1	4±2	4±3	0.538†
Bloating (mm)	24±8	25±7	34±9	0.859†
Flatulence (mm)	28±7	46±9	45±10	0.190†
Heartburn (mm)	13±5	8±6	12±8	0.773†
Belching (mm)	2±1	6±4	3±1	0.401†

Data are expressed as mean ± SEM (n=12). Energy intake was recorded with 3-day food diaries. Fibre defined as non-starch polysaccharide. Physical activity was measured using the short self-administered format of the IPAQ [11]. Ratings of gastrointestinal side-effects were made using 100 mm visual analogue scales (VAS). Subjects were asked to rate the occurrence of each side effect with extreme statements anchored at each end of the rating scale (0 mm *Never*, 100 mm *All the time*). P value is from repeated measures ANOVA. †P value from Friedman test.

Suppl. Table 5. Fasting and postprandial metabolic responses following 42 days of cellulose, inulin and inulin propionate ester (IPE) supplementation.

	Cellulose	Inulin	IPE	P Value
**Fasting Glucose (mmol/L)**	**5.3±0.1**	**5.3±0.2**	**5.2±0.2**	**0.852**
**Postprandial Glucose (mmol/L×min)**	**1187±56**	**1159±64**	**1149±61**	**0.475**
**Fasting Insulin (µU/mL)**	**12.3±1.4**	**9.0±1.2****	**9.4±1.2^^**	**0.001**
**Postprandial Insulin (µU/mL×min)**	**18916±2234**	**18478±2182**	**17711±2354**	**0.575**
**Fastng NEFA (µmol/L)**	**701±068**	**648±070**	**681±039**	**0.613**
**Postprandial NEFA (µmol/L×min)**	**31014±1799**	**31200±10557**	**32091±1791**	**0.892**
**Fastng PYY (pmol/L)**	**31.9±5.5**	**30.5±5.2**	**29.4±5.2**	**0.307**
**Postprandial PYY (pmol/L×min)**	**8414±633**	**8002±756**	**7941±694**	**0.369**
**Fastng Active GLP-1 (pmol/L)**	**13.2±2.0**	**13.4±2.4**	**13.9±2.4**	**0.706**
**Postprandial Active GLP-1 (pmol/L×min)**	**3042±346**	**2895±367**	**2957±362**	**0.417**
**HbA1c (mmol/mol)**	**36.3±1.1**	**36.3±1.2**	**35.8±1.0**	**0.657**
**Triglycerides (mmol/L)**	**1.1±0.1**	**1.0±0.1**	**1.0±0.1**	**0.457**[†]
**Cholesterol (mmol/L)**	**5.2±0.3**	**5.3±0.2**	**5.2±0.3**	**0.642**
**LDL Cholesterol (mmol/L)**	**3.3±0.2**	**3.3±0.1**	**3.2±0.2**	**0.950**
**HDL Cholesterol (mmol/L)**	**1.5±0.1**	**1.6±0.1**	**1.5±0.1**	**0.243**
**Alanine Transaminase (IU/L)**	**23.0±3.9**	**21.5±1.8**	**24.2±2.5**	**0.480**
HOMA2-IR	**1.6±0.2**	**1.2±0.2****	**1.2±0.2^^**	**0.001**
Matsuda ISI	**3.2±0.5**	**4.0±0.7***	**4.0±0.6^^**	**0.003**[†]
***AT-IR* (µU/ml × mmol/L)**	**8.3±1.3**	**6.3±1.5***	**6.5±1.0^**	**0.046**[†]
Oral Disposition Index	**10.2±0.8**	**16.2±3.2***	**13.7±1.9^**	**0.005**[†]

Postprandial values calculated as AUC. Data are expressed as mean ± SEM (n=12). *= P<0.05, **=P<0.01 cellulose vs. inulin. ^=p<0.05, ^^=p<0.01 cellulose vs. IPE. P value is from repeated measures ANOVA. [†]P value from Friedman test.

Suppl. Table 6. The antibody panel used to analyse the cell surface phenotype of PBMCs

Antibody	Fluorochrome	Clone	Supplier
CD8	AF700	RPA-T8	Becton Dickinson
CD4	FITC	RPA-T4	Becton Dickinson
CD3	APC-Cy7	SK7	Becton Dickinson
CD19	PerCP-Cy5.5	HIB19	Biolegend
CD183	PE	1C6/CXCR3	Becton Dickinson
CD196	BV605	11A9	Becton Dickinson
CD194	AF647	205410	R&D Systems
CD25	BV421	BC96	Biolegend
CD127	PE-CF594	HIL-7R-M21	Becton Dickinson
CD45RO	PE-Cy5	UCHL1	Becton Dickinson
Live/Dead	FVS510	-	Becton Dickinson

Suppl. Table 7. Immunoglobulin (Ig) and inflammatory markers following 42 days of cellulose, inulin and inulin propionate ester (IPE) supplementation.

	Cellulose	Inulin	IPE	P Value
IgA (g/L)	2.52±0.26	2.55±0.26	2.60±0.28	0.195
IgG (g/L)	9.89±0.38	10.20±0.50	10.29±0.45^^	0.007
IgM (g/L)	0.82±0.11	0.81±0.10	0.82±0.10	0.760
C Reactive Protein (mg/L)	2.24±0.71	1.69±0.47	1.84±0.34	0.656
IL-6 (pg/mL)	2.27±0.77	2.68±0.80	1.75±0.38	0.472†
IL-8 (pg/mL)	8.69±1.74	8.05±1.36	5.86±0.59^	0.017†
IL-10 (pg/mL)	0.25±0.07	0.36±0.08	0.26±0.08	0.240
IL-12 (pg/mL)	24.47±4.13	23.96±2.66	20.81±3.28	0.969
LBP (ng/mL)	12.25±0.79	11.44±0.73	11.35±0.87	0.368

Data are expressed as mean ± SEM (n=12). ^=p<0.05, ^^=p<0.01 cellulose vs. IPE. P value is from repeated measures ANOVA. †P value from Friedman test.

SUPPLEMENTARY FIGURES

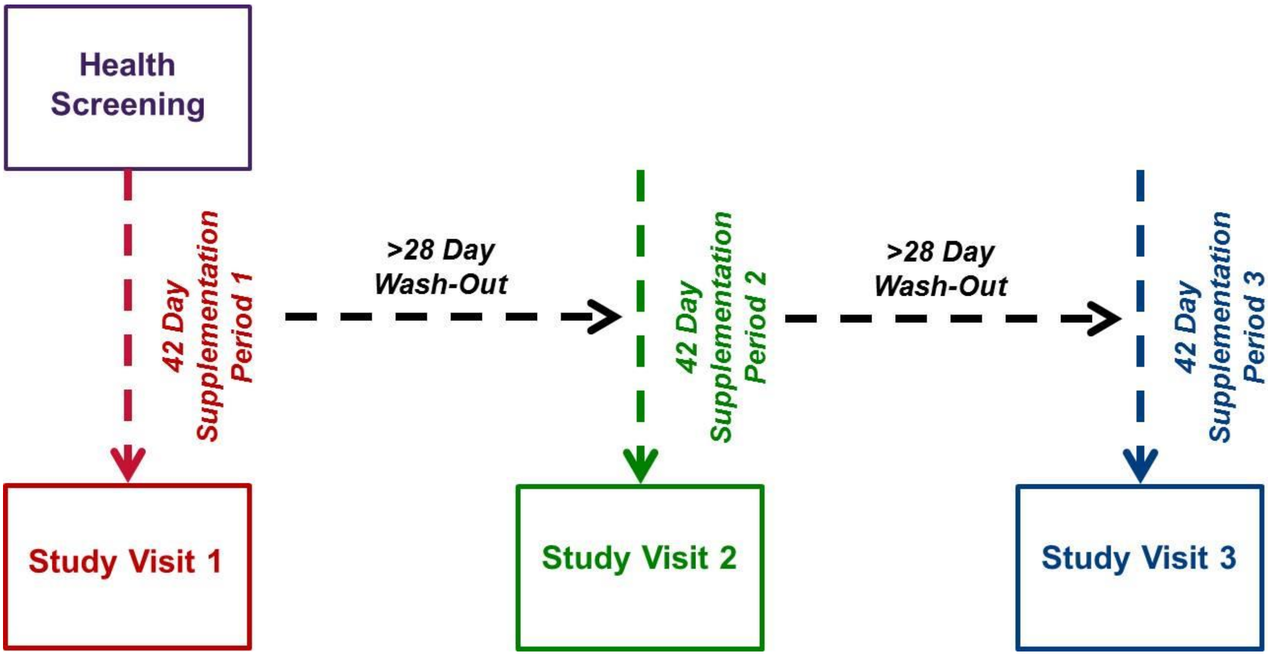

Suppl. Figure 1. A summary of the randomised, double-blind, placebo controlled crossover design.

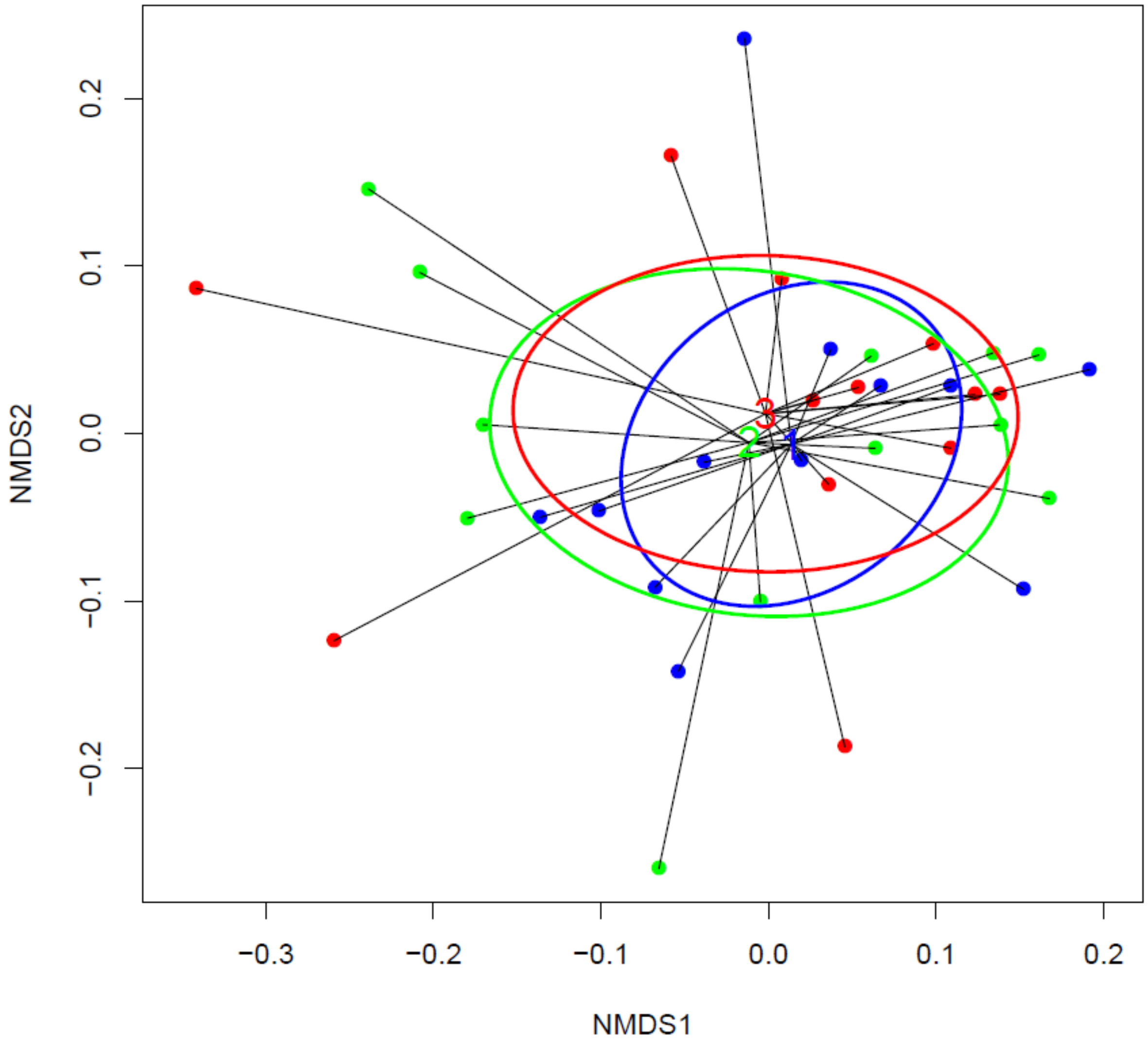

Suppl. Figure 2. The effect of supplementation order on the gut microbiota. Nonmetric multidimensional scaling (NMDS) plot showing no differences in the microbial composition of faecal samples collected after supplementation period 1 (blue), 2 (green) and 3 (red) ($p > 0.05$ for all comparisons, PERMANOVA).

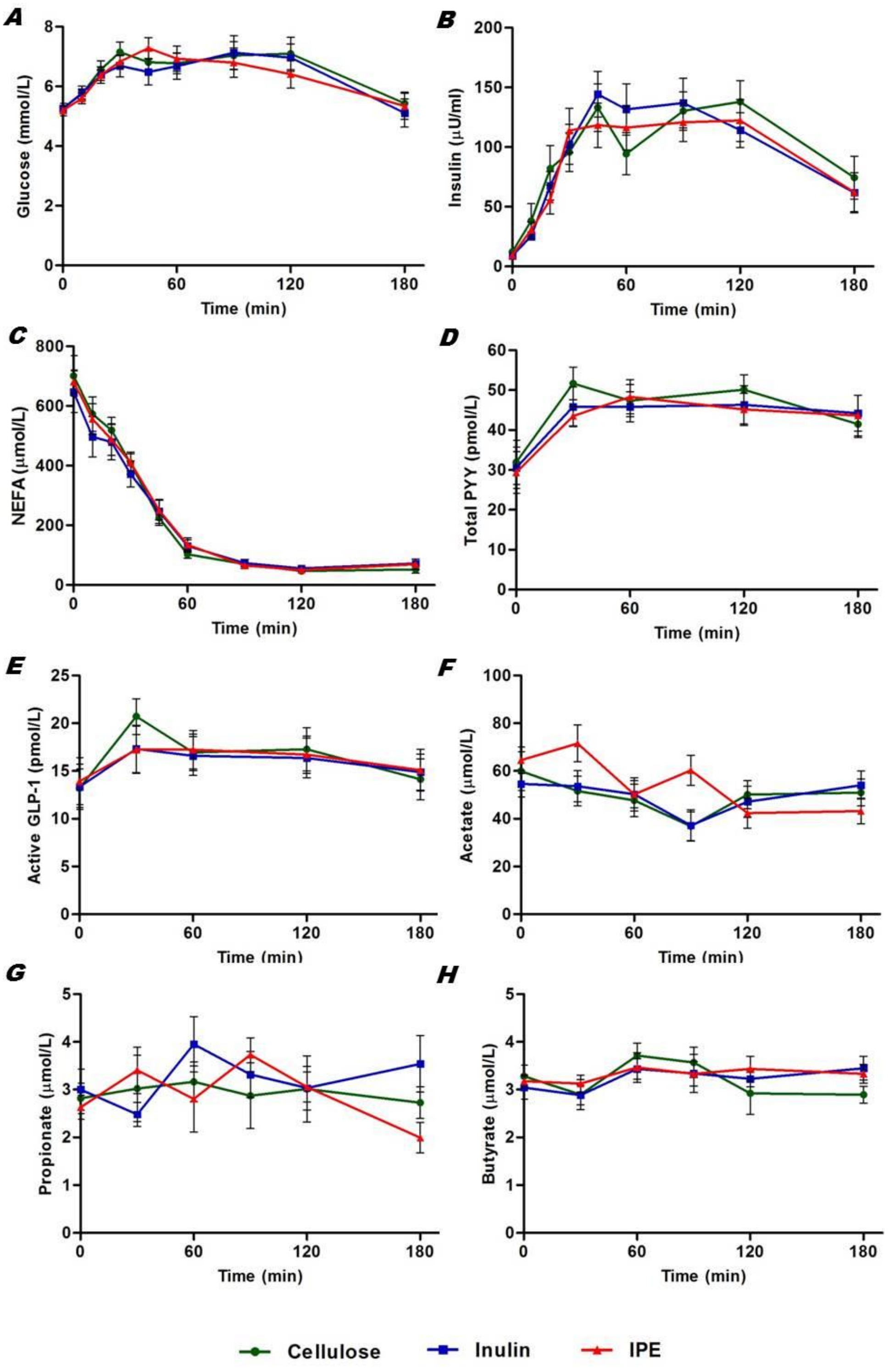

Suppl. Figure S3. The effects of 42 days of cellulose, inulin and inulin propionate ester (IPE) supplementation on postprandial metabolic responses. ***A.*** Plasma Glucose, ***B.***, ***C.*** Serum NEFA, ***D.*** Plasma Total PYY, ***E.*** Plasma Active GLP-1, ***F.*** Serum Acetate, ***G.*** Serum Propionate, and ***H.*** Serum Butyrate. Data are expressed as mean ± SEM (n = 12).

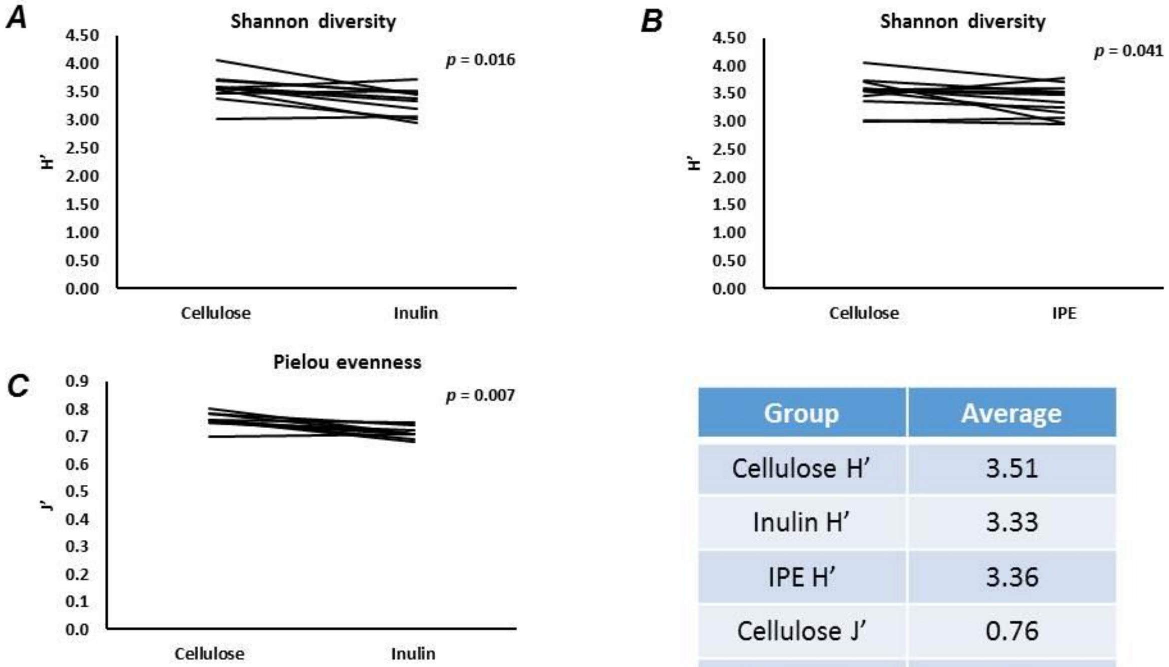

Group	Average
Cellulose H’	3.51
Inulin H’	3.33
IPE H’	3.36
Cellulose J’	0.76
Inulin J’	0.71

Suppl. Figure 4: The effects of 42 days of cellulose, inulin and inulin propionate ester (IPE) supplementation on gut bacterial diversity and evenness. n = 12, p values from Wilcoxon signed rank test. H’ – Shannon diversity index, J’ = Pielou’s evenness index.

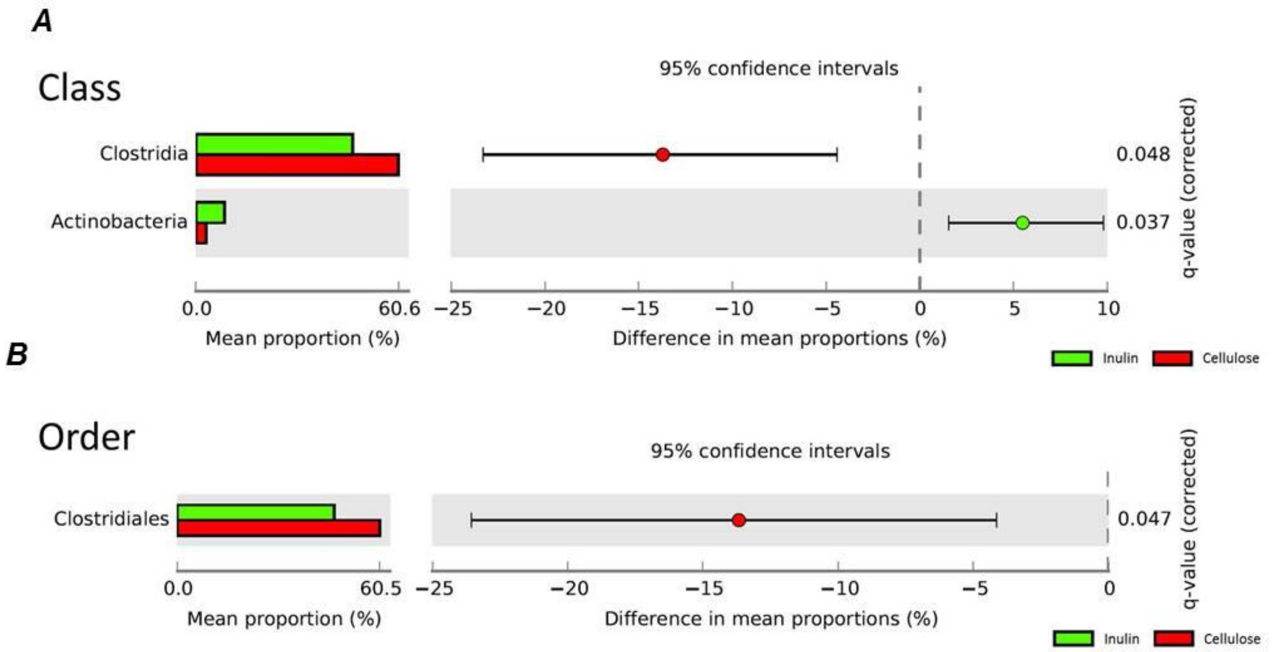

Suppl. Figure 5. Changes in class *A.* and *B.* order of bacterial species following the 42 day inulin and cellulose supplementation periods. No significant differences were observed between the inulin and inulin-propionate ester (IPE) supplementation periods and the IPE and cellulose supplementation periods. n = 12, p values from Wilcoxon signed rank test.

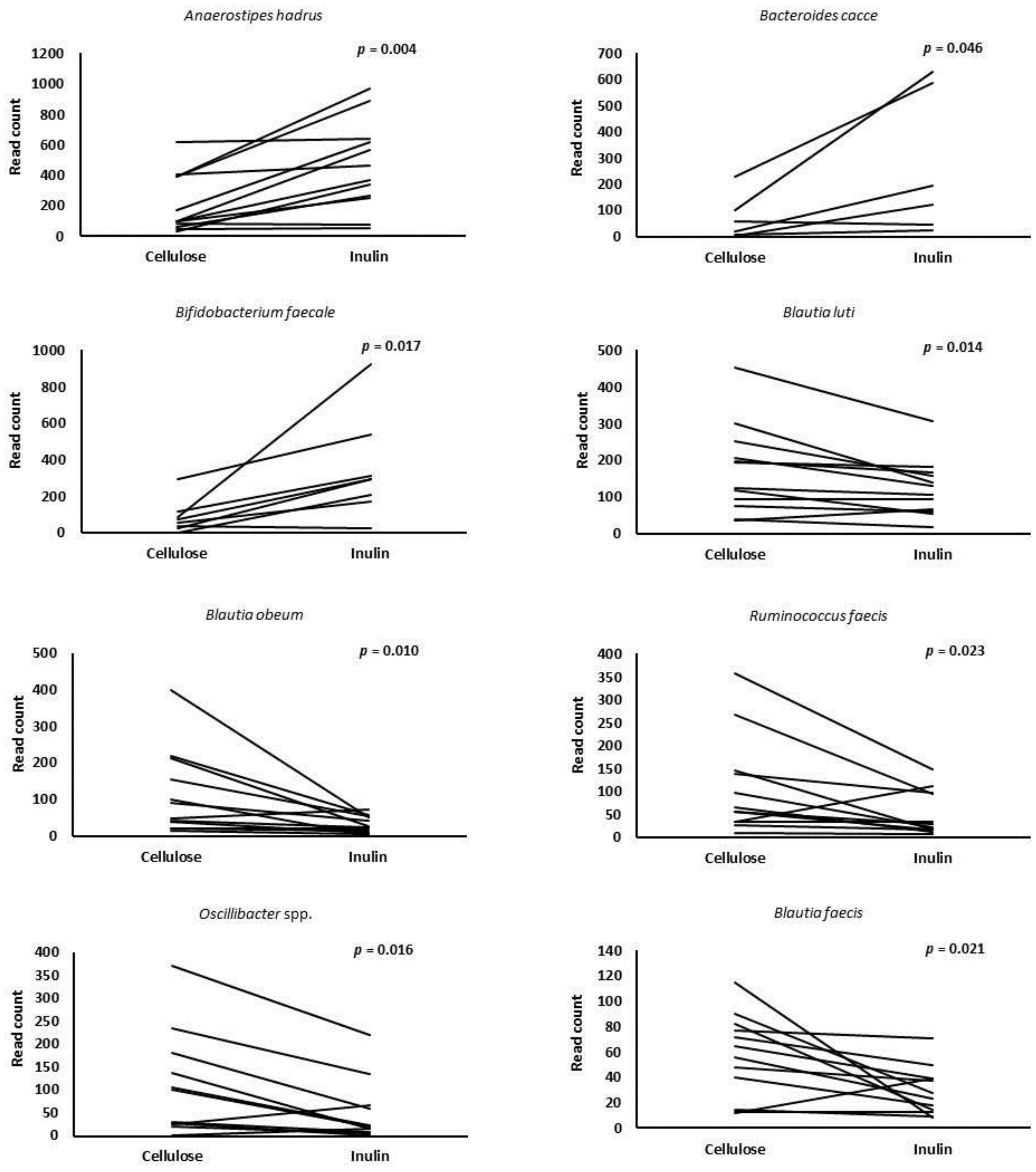

Suppl. Figure 6. Changes in bacterial 16S rRNA gene sequencing read counts following the 42 day cellulose and inulin supplementation periods. n = 12, P values from Wilcoxon signed rank test.

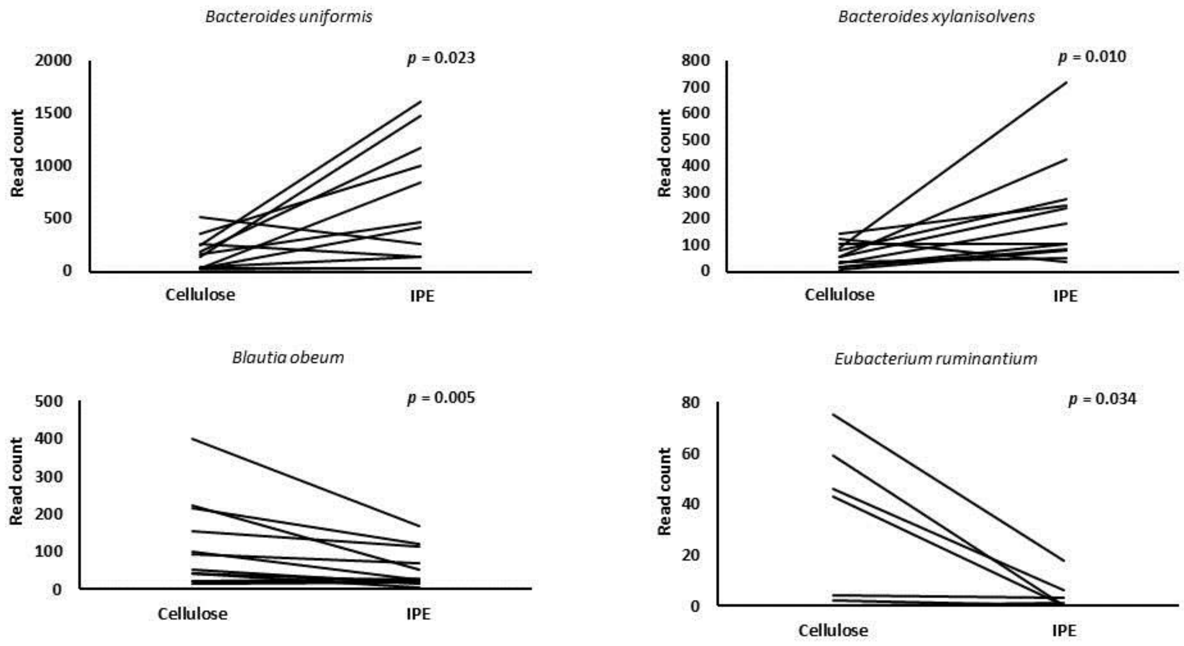

Suppl. Figure 7. Changes in bacterial 16S rRNA gene sequencing read counts following the 42 day cellulose and inulin-propionate ester (IPE) supplementation periods. n = 12, P values from Wilcoxon signed rank test.

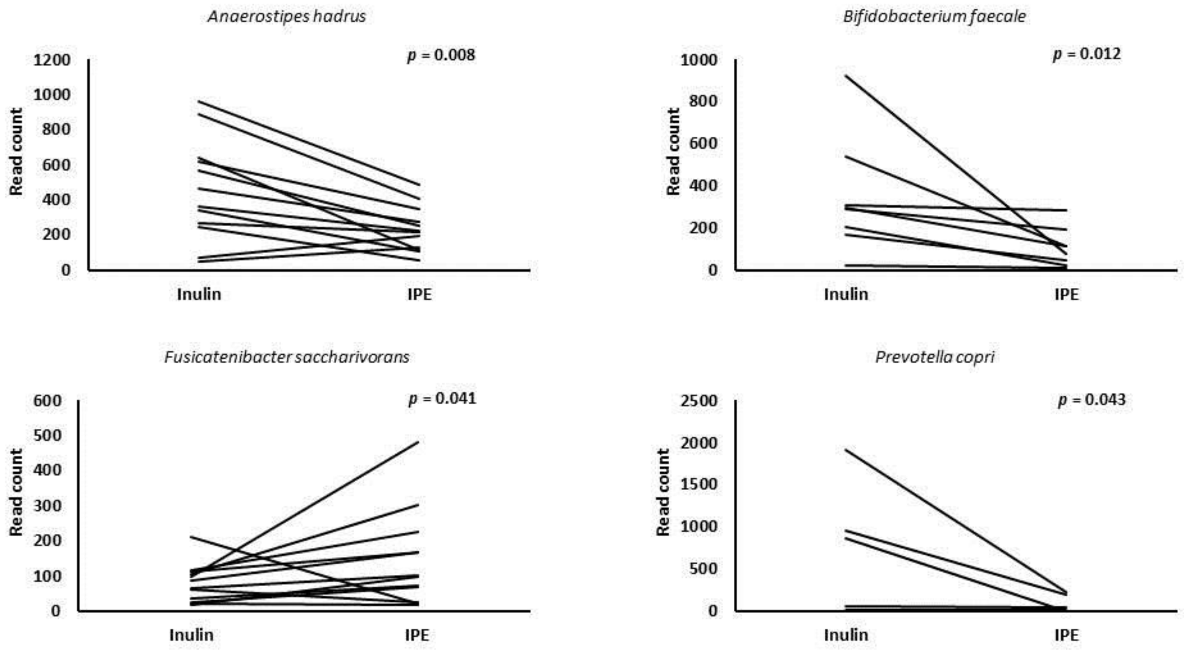

Suppl. Figure 8. Changes in bacterial 16S rRNA gene sequencing read counts following the 42 day inulin and inulin-propionate ester (IPE) supplementation periods. n = 12, P values from Wilcoxon signed rank test.

REFERENCES

1. Moreau NM, Goupry SM, Antignac JP, Monteau FJ, Le Bizec BJ, Champ MM, et al. Simultaneous measurement of plasma concentrations and 13C-enrichment of short-chain fatty acids, lactic acid and ketone bodies by gas chromatography coupled to mass spectrometry. *J Chromatogr B Analyt Technol Biomed Life Sci* 2003;784(2):395-403.
2. Dona AC, Jimenez B, Schafer H, Humpfer E, Spraul M, Lewis MR, et al. Precision high-throughput proton NMR spectroscopy of human urine, serum, and plasma for large-scale metabolic phenotyping. *Anal Chem* 2014;86(19):9887-94.
3. Dieterle F, Ross A, Schlotterbeck G, Senn H. Probabilistic quotient normalization as robust method to account for dilution of complex biological mixtures. Application in 1H NMR metabonomics. *Anal Chem* 2006;78(13):4281-90.
4. Posma JM, Garcia-Perez I, Ebbels TMD, Lindon JC, Stamler J, Elliott P, et al. Optimized Phenotypic Biomarker Discovery and Confounder Elimination via Covariate-Adjusted Projection to Latent Structures from Metabolic Spectroscopy Data. *J Proteome Res* 2018;17(4):1586-95.
5. Garcia-Perez I, Posma JM, Gibson R, Chambers ES, Hansen TH, Vestergaard H, et al. Objective assessment of dietary patterns by use of metabolic phenotyping: a randomised, controlled, crossover trial. *Lancet Diabetes Endocrinol* 2017;5(3):184-95.
6. Posma JM, Garcia-Perez I, De Iorio M, Lindon JC, Elliott P, Holmes E, et al. Subset optimization by reference matching (STORM): an optimized statistical approach for recovery of metabolic biomarker structural information from 1H NMR spectra of biofluids. *Anal Chem* 2012;84(24):10694-701.
7. Quigley KJ, Reynolds CJ, Goudet A, Raynsford EJ, Sergeant R, Quigley A, et al. Chronic Infection by Mucoid Pseudomonas aeruginosa Associated with Dysregulation in T-Cell Immunity to Outer Membrane Porin F. *Am J Respir Crit Care Med* 2015;191(11):1250-64.
8. Kozich JJ, Westcott SL, Baxter NT, Highlander SK, Schloss PD. Development of a dual-index sequencing strategy and curation pipeline for analyzing amplicon sequence data on the MiSeq Illumina sequencing platform. *Appl Environ Microbiol* 2013;79(17):5112-20.
9. Wang Q, Garrity GM, Tiedje JM, Cole JR. Naive Bayesian classifier for rapid assignment of rRNA sequences into the new bacterial taxonomy. *Appl Environ Microbiol* 2007;73(16):5261-7.
10. Garcia-Villalba R, Gimenez-Bastida JA, Garcia-Conesa MT, Tomas-Barberan FA, Carlos Espin J, Larrosa M. Alternative method for gas chromatography-mass spectrometry analysis of short-chain fatty acids in faecal samples. *J Sep Sci* 2012;35(15):1906-13.
11. Craig CL, Marshall AL, Sjostrom M, Bauman AE, Booth ML, Ainsworth BE, et al. International physical activity questionnaire: 12-country reliability and validity. *Med Sci Sports Exerc* 2003;35(8):1381-95.
12. Chambers ES, Viardot A, Psichas A, Morrison DJ, Murphy KG, Zac-Varghese SE, et al. Effects of targeted delivery of propionate to the human colon on appetite regulation, body weight maintenance and adiposity in overweight adults. *Gut* 2015;64(11):1744-54.
13. Robertson MD, Wright JW, Loizon E, Debard C, Vidal H, Shojaee-Moradie F, et al. Insulin-sensitizing effects on muscle and adipose tissue after dietary fiber intake in men and women with metabolic syndrome. *J Clin Endocrinol Metab* 2012;97(9):3326-32.
14. Matthews DR, Hosker JP, Rudenski AS, Naylor BA, Treacher DF, Turner RC. Homeostasis model assessment: insulin resistance and beta-cell function from fasting plasma glucose and insulin concentrations in man. *Diabetologia* 1985;28(7):412-9.
15. Levy JC, Matthews DR, Hermans MP. Correct homeostasis model assessment (HOMA) evaluation uses the computer program. *Diabetes Care* 1998;21(12):2191-2.
16. Matsuda M, DeFronzo RA. Insulin sensitivity indices obtained from oral glucose tolerance testing: comparison with the euglycemic insulin clamp. *Diabetes Care* 1999;22(9):1462-70.
17. Lomonaco R, Ortiz-Lopez C, Orsak B, Webb A, Hardies J, Darland C, et al. Effect of adipose tissue insulin resistance on metabolic parameters and liver histology in obese patients with nonalcoholic fatty liver disease. *Hepatology* 2012;55(5):1389-97.

18. Retnakaran R, Qi Y, Goran MI, Hamilton JK. Evaluation of proposed oral disposition index measures in relation to the actual disposition index. *Diabet Med* 2009;26(12):1198-203.
